# Sensitive Detection of Viral Transcripts in Human Tumor Transcriptomes

**DOI:** 10.1371/journal.pcbi.1003228

**Published:** 2013-10-03

**Authors:** Sven-Eric Schelhorn, Matthias Fischer, Laura Tolosi, Janine Altmüller, Peter Nürnberg, Herbert Pfister, Thomas Lengauer, Frank Berthold

**Affiliations:** 1 Max-Planck-Institute for Informatics, Campus E1.4, Saarbrücken, Germany; 2 Department of Pediatric Oncology and Hematology, Children's Hospital, and Center for Molecular Medicine Cologne, University of Cologne, Cologne, Germany; 3 Cologne Center for Genomics, University of Cologne, Cologne, Germany; 4 Institute of Virology, University of Cologne, Cologne, Germany; University of Texas at Austin, United States of America

## Abstract

In excess of 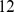
% of human cancer incidents have a viral cofactor. Epidemiological studies of idiopathic human cancers indicate that additional tumor viruses remain to be discovered. Recent advances in sequencing technology have enabled systematic screenings of human tumor transcriptomes for viral transcripts. However, technical problems such as low abundances of viral transcripts in large volumes of sequencing data, viral sequence divergence, and homology between viral and human factors significantly confound identification of tumor viruses. We have developed a novel computational approach for detecting viral transcripts in human cancers that takes the aforementioned confounding factors into account and is applicable to a wide variety of viruses and tumors. We apply the approach to conducting the first systematic search for viruses in neuroblastoma, the most common cancer in infancy. The diverse clinical progression of this disease as well as related epidemiological and virological findings are highly suggestive of a pathogenic cofactor. However, a viral etiology of neuroblastoma is currently contested. We mapped 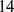 transcriptomes of neuroblastoma as well as positive and negative controls to the human and all known viral genomes in order to detect both known and unknown viruses. Analysis of controls, comparisons with related methods, and statistical estimates demonstrate the high sensitivity of our approach. Detailed investigation of putative viral transcripts within neuroblastoma samples did not provide evidence for the existence of any known human viruses. Likewise, *de-novo* assembly and analysis of chimeric transcripts did not result in expression signatures associated with novel human pathogens. While confounding factors such as sample dilution or viral clearance in progressed tumors may mask viral cofactors in the data, in principle, this is rendered less likely by the high sensitivity of our approach and the number of biological replicates analyzed. Therefore, our results suggest that frequent viral cofactors of metastatic neuroblastoma are unlikely.

## Author Summary

Many human cancers are caused by infections with tumor viruses and identification of these pathogens is considered a critical contribution to cancer prevention. Deep sequencing enables us to systematically investigate viral nucleotide signatures in order to either verify or exclude the existence of viruses in idiopathic human cancers. We have developed Virana, a novel computational approach for identifying tumor viruses in human cancers that is applicable to a wide variety of tumors and viruses. Virana firstly addresses several important biological confounding factors that may hinder successful detection of these pathogens. We applied our approach in the first systematic search for cancer-causing viruses in metastatic neuroblastoma, the most common form of cancer in infancy. Although the heterogeneous clinical progression of this disease as well as epidemiological and virological findings are suggestive of a pathogenic cofactor, the viral etiology of neuroblastoma is currently contested. We conducted an analysis of experimental controls, comparisons with related approaches, as well as statistical analyses in order to validate our method. In spite of the high sensitivity of our approach, analyses of neuroblastoma transcriptomes did not provide evidence for the existence of any known or unknown human viruses. Our results therefore suggest that frequent viral cofactors of metastatic neuroblastoma are unlikely.

## Introduction

To date, pathogenic agents are known to be causally related to 20% of human cancer cases [1] and significantly affect the global health burden of this disease [2]. The majority of these agents comprise oncogenic viruses such as human papilloma virus (HPV), Epstein-Barr virus (EBV), hepatitis B virus (HBV), and hepatitis C virus (HCV) [3]. Characterizing the oncogenic potential of viral pathogens has important consequences for prevention, diagnosis, and treatment of malignant neoplasms [4], [5]. Tumor viruses in particular have received renewed attention in the context of recent global efforts to characterize the etiology of cancer [6], [7]. Consequently, viral cofactors for several idiopathic cancers are currently investigated [8] and epidemiological indicators suggest that additional human tumor viruses remain to be discovered [9].

Neuroblastoma is a heterogeneous embryonal tumor [10], [11] that is accountable for 15% of deaths caused by malignant conditions in children [12]. The disease is associated with an exceptionally low median age of presentation of 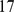 months [13] and is often diagnosed *in utero*. Metastatic neuroblastoma has two biologically divergent subtypes. Stage 4S is characterized by an age of presentation between *in utero* and 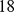 months, metastases confined to liver, skin, lymph nodes and bone marrow, and its ability to regress spontaneously [14], [15]. In contrast, stage 4 tumors are presented at any age, demonstrate high infiltration rates in bone marrow and bone, and are most often progressive [10], [16]. While genes related to neuronal differentiation have been described to be upregulated in stage 4S in comparison to stage 4 neuroblastoma, thereby indicating distinct levels of neuronal differentiation [17], little is currently known about the differences between molecular etiologies of stage 4 and stage 4S neuroblastoma.

The variation of clinical outcomes between neuroblastoma subtypes indicates distinct genetic and environmental factors affecting the development of this malignancy. Interestingly, the early onset of the disease overlaps with periods of high susceptibility to viral infections and is reminiscent of acute lymphoblastic leukemia – another pediatric tumor with uncertain etiology for which an infective cofactor has long been suspected [18]. Furthermore, epidemiological studies have associated reduced neuroblastoma risk with immunologic indicators such as previous childhood infections, day care attendance, and breast feeding [19], [20] that are suggestive of an infective cofactor [21]. While transforming polyomaviruses such as JCV and BKV were previously identified within neuroblastoma samples and other pediatric embryonal tumors [22]–[24], newer studies seem to render these associations inconclusive [25]. Therefore, the role of pathogenic cofactors of neuroblastoma oncogenesis remains unresolved.

In general, the search for suspected viral cofactors of idiopathic diseases requires systematic screening of human tissues for viral biomarkers such as virus-derived nucleotide sequences. Unfortunately, viruses are of polyphyletic origin and thus lack common universal marker genes as they are frequently exploited in metagenomics studies targeting cellular microorganisms. Consequently, it is not currently possible to specifically PCR-amplify viral nucleotide sequences within a given tissue without prior information about the infective agent being sought [26]. As a result, several systematic assays for pathogen detection have been developed that do not rely on targeted PCR-amplification of viral factors [27] and were employed to identify Kaposi's sarcoma-associated herpes virus (KSHV) as a human tumor virus [28]. These systematic approaches were recently supplemented by sensitive deep sequencing technologies [27]. These technologies were recently applied to exclude several cancer-virus associations based on negative evidence [29], [30] and aided in the identification of MCPyV, a human polyomavirus, as a cofactor of Merkel cell carcinoma [31].

Deep sequencing technologies have enabled detection of both known and novel viruses with unprecedented sensitivity [32]. However, the large numbers of sequence fragments (“reads”) generated by these methods necessitate data reduction approaches for filtering and condensing the list of putative viral transcripts. Two such approaches are currently represented in the literature: *digital transcript subtraction* that discards human sequence homologs from the sequence data and considers the remaining transcripts as potential viral signatures [30], [31], [33]–[39], and *de-novo sequence assembly* that aims to reconstruct whole viral genomes from overlapping reads [40]–[43]. Recently, variants of these of two approaches have been implemented in several computational pipelines such as PathSeq [44], RINS [45], and CaPSID [46].

Identification of tumor viruses in particular poses several important challenges to existing computational pipelines. Confounding factors such as loss of viral genetic material from progressed tumors as well as limited replication competence or latent replication strategies often result in low or selective transcription of tumor viruses [5]. In addition, viral oncogenes homologous to human factors and chimeric transcripts originating from proviral insertion sites may share significant sequence similarity with human transcripts [47], thus making unequivocal identification of viral factors difficult. Last, high rates of viral sequence divergence from 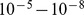 (dsDNA viruses) up to 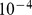 (ssRNA viruses) substitutions per site and year [48], [49] hinder recognition of known viruses based on known reference sequences.

We have developed Virana, a novel computational approach specifically tailored to detecting low-abundance transcripts that diverge from known viral reference sequences or share significant sequence homology with human factors. In particular, our method maps sequence reads to a combined reference database comprising the human genome and all known viral reference sequences. The approach is configured to allow for high mismatch rates and mappings to multiple reference sequences (‘*multimaps’*). By using this combined and sensitive mapping strategy, our approach is especially well suited for detecting human-viral chimeric transcripts and viruses diverging from known references. In contrast to existing subtractive approaches for viral transcript discovery, our method abstains from discarding reads homologous to the human genome from further analysis. Instead, Virana exploits multimaps to assign sequence reads to a homologous context comprising human reference transcripts and viral reference genomes. These homologous regions retain the full, unfiltered information contained in the raw sequence data while also being amenable to further analyses by multiple sequence alignments, human-viral phylogenies, and orthogonal taxonomic annotations, thus greatly aiding in the interpretation of the results.

We applied our novel approach on an overall number of 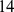 deep sequencing transcriptomes of stage 4 and stage 4S metastatic neuroblastoma in order to identify putative viral cofactors associated with this idiopathic disease.

## Materials and Methods

### Clinical samples and experimental deep sequencing data

Primary neuroblastoma samples from stage 4 (progressive) patients (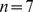) and stage 4S (regressive) patients (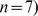 were obtained prior to treatment from the central neuroblastoma tumor bank at the University Hospital of Cologne, Germany. None of the tumors harbored amplification of the MYCN proto-oncogene as determined by two independent laboratories for each case by fluorescence in situ hybridisation (FISH) and Southern blot [50]. Only neuroblastoma samples with a tumor cell content of above 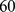
% as assessed by a pathologist were selected for deep sequencing. Integrity of RNA was evaluated using the Bioanalyzer 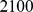 (Agilent Technologies) and only samples with an RNA integrity number of at least 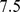 were considered for further processing. Quality of all neuroblastoma samples and related deep sequencing data was additionally confirmed by an orthogonal computational analysis focusing on human gene expression in the context of differential splicing [51].

All patients were enrolled in the German Neuroblastoma trials with informed consent. In order to validate our approach we additionally employed a positive control panel consisting of tumors with known viral cofactors. An EBV-positive B-cell-lymphoma (BCL) was received from the Pediatric Oncology and Hematology Department of the Hannover Medical School. Deep-sequencing reads obtained from full transcriptome libraries of two HPV18-positive HeLa samples (HeLa) and a HPV16-positive primary cervical squamous cell carcinoma (ceSCC) were downloaded from the Short Read Archive (SRA) and preprocessed as specified in the original publication [30]. Transcriptome data of a HBV-positive hepatocellular carcinoma (HCC) HKCI-5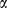 cell line with confirmed HBV integration events was downloaded from the SRA based on information in the original publication [52]. A negative control panel consisting of a normal brain transcriptome generated as part of the Illumina BodyMap 2.0 project was obtained from the SRA at run accession number ERR030882.

### Library preparation and sequencing

mRNA libraries of the EBV-positive B-cell lymphoma and 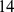 neuroblastomas were prepared following the Illumina RNA Sample Preparation Kit and Guide (Part #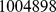 Rev. A). For each sample, 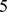 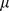
g high-quality total RNA was processed for mRNA purification, chemical fragmentation, first strand synthesis, second strand synthesis, end repair, 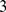
′-end adenylation, adapter ligation, and PCR amplification. Validated libraries underwent gel size selection and final paired-end sequencing with an effective read length of 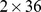 bp on the Illumina Genome Analyzer IIx following Illumina standard protocols. Additionally, libraries for two of the 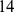 neuroblastoma samples were generated using the same protocols and sequenced with an effective paired-end read length of 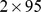 bp on a Illumina HiSeq 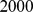. All libraries had insert size distributions approximating 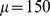 bp, 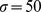 bp as later confirmed by read mapping. The data were filtered according to signal purity by the Illumina Realtime Analysis (RTA) software.

### Simulated sequencing data

In this study we employ simulated sequencing data from three viral genomes that are homologous to human factors. Reads originating from the ABL1-homologue of the Abelson murine leukemia virus (A-MuLV, GI:9626953, positions 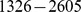), from the the *gag* region of HERVK22I (obtained from Repbase [53], positions 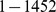), and from Bo17, a GCNT3-homolog of the bovine herpesvirus 4 (BoHV-4, GI:13095578, positions 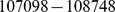) were generated *in silico* by dwgsim, a read simulator based on wgsim [54]. In addition, we produced simulated chimeric transcripts by fusing each of the aforementioned sequence regions to the human TP53 gene, a known proto-oncogene (UCSC build hg19, GRCh37, chr17, positions 7572926–7579569). These artificial fusion transcripts were generated using Fusim [55] based on TP53 exon models obtained from the UCSC refGene database [56]. Fusion transcripts were then used as templates for generating simulated data sets with dwgsim. In all cases, dwgsim was applied using the default empirical error model. Paired-end read lengths and insert size distributions were chosen according to the neuroblastoma sequencing data (see above). Additional simulated sequencing data generated by a related publication were analyzed as described in Section “Estimation of read mapping sensitivity”.

### Sample data notation

Sample panels containing neuroblastoma transcriptomes sequenced at 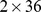 bp and 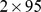 bp effective read lengths are denoted as NB1 and NB2, respectively. While the NB1 panel contains seven transcriptomes of neuroblastoma stages 4 and 4S each, the NB2 panel contains one sample of stages 4 and 4S each (see Table 1). Positive control panels of human cancer transcriptomes with known viral cofactors (BCL, HeLa, ceSCC, and HCC) are denoted as POS. The negative control panel consisting of a normal human brain transcriptome is denoted as NEG.

**Table 1:** Sequencing panel characteristics.

Panel	Source	Sample ID	Read length	Sequencing depth (Gbp)	Read pairs (million)
POS	HeLa	15	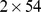 bp	0.076	0.737
POS	ceSCC	16	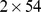 bp	0.157	1.527
POS	ceSCC	17	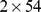 bp	0.041	0.400
POS	BCL	18	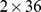 bp	3.134	43.527
POS	HCC	19	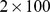 bp	11.22	55.547
NEG	Brain	20	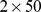 bp	7.351	73.513
NB1	4	1	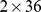 bp	1.184	16.439
NB1	4	2	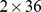 bp	0.770	10.695
NB1	4	3	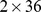 bp	0.881	12.236
NB1	4	4	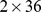 bp	0.744	10.345
NB1	4	5	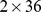 bp	1.207	16.759
NB1	4	6	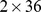 bp	1.050	14.581
NB1	4	7	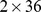 bp	0.829	11.527
NB1	4S	8	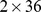 bp	1.031	14.317
NB1	4S	9	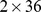 bp	1.172	16.282
NB1	4S	10	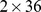 bp	0.868	12.065
NB1	4S	11	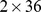 bp	0.890	12.368
NB1	4S	12	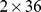 bp	0.845	11.737
NB1	4S	13	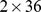 bp	1.174	16.300
NB1	4S	14	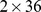 bp	0.847	11.772
NB2	4	7	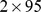 bp	9.284	48.863
NB2	4S	13	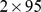 bp	8.748	46.041

Sequencing characteristics of neuroblastoma (NB), positive control (POS), and negative control (NEG) panels.

### Reference genomes

The current assembly of the human reference genome (UCSC build hg19, GRCh37) as well as corresponding refGene splice-site annotations were obtained from UCSC. Splice variant annotations and cDNA sequences for the human genome were downloaded from Ensembl [57]. A set of all 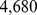 available complete viral reference genomes and their taxonomic lineages were obtained from NCBI via the E-utilities web service [58] and the database query: “Viruses[Organism] AND srcdb_refseq[PROP] NOT cellular organisms [ORGN]”. In addition, we obtained consensus reference sequences for all human endogenous retroviruses (HERV-K/HML-2) represented in Repbase (Primate HERV, HERVK11DI, HERVK11I, HERVK13I, HERVK22I, HERVK3I, HERVK9I, HERVKC4)) [53]. All reference genomes were combined into a single human-viral reference database for Virana. Since RINS and CaPSID cannot use such a combined database, human and viral reference sequences were collected within two separate databases for these approaches.

### Quality control, mapping, and assembly

Paired-end reads from the neuroblastoma panels and positive control panels were quality-controlled with an in-house sequence analysis framework in order to identify sample contamination, adapter contamination, and batch effects. After quality control, the sequence data consisted of 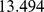 Gbp (NB1), 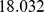 Gbp (NB2), 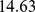 Gbp (POS), and 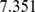 Gbp (NEG) of sequence reads, respectively (see Table 1).

All data were mapped against a combined human-viral reference database with the splicing-aware and gapped read mapper STAR [59] in paired-end mode. While Virana considers the read mapper to be a replaceable component, in principle, we decided to employ STAR due to its mapping speed, high sensitivity settings, and its consideration of putative chimeric transcripts. We configured the mapper for high sensitivity by following recommendations of the author of STAR (personal communication). In particular, we set the rate of acceptable mismatches to 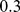 times the length of each read and the *seedSearchStartLmax* and *winAnchorMultimapNmax* parameters to 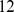 and 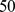, respectively. The minimum length of chimeric segments (*chimSegmentMin*) was reduced to 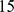 in order to detect fusion transcripts at short read lengths. Known splice sites from splice annotations of the human reference genome as well as canonical splice sites were considered in the mapping. For each read, multiple mapping locations with alignment score distances of up to 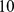 ranks relative to the best score were permitted (‘multimaps’). Read alignments were stored in standardized BAM files. STAR supports detection of chimeric transcripts by reporting discordant read pairs whose ends map to different chromosomes. These discordant read pairs were employed in further analyses as detailed in the next section.

In order to identify putative new viral transcripts, read pairs with at least one unmapped read end were extracted from BAM files by the Samtools suite [54] and assembled into longer contigs by the *de-novo* transcriptome assemblers Trinity [60] and Oases [61] using default parameters. Oases was configured for using different k-mer values in order to facilitate reconstruction of low-abundance viral transcripts. Contigs of length less than 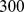 bp were considered to be spurious assemblies and excluded from further processing.

### Detection of chimeric transcripts

Virana supports detection of human-viral chimeric transcripts in two different manners. First, the read mapper employed in our study is able to partially align reads that contain a human-viral chimeric breakpoint to multiple reference sequences. Consequently, these partially aligned reads can be detected by Virana within the generic analysis of homologous regions (see below). The second, more sensitive approach to detecting chimeric transcripts is based on paired-end read information. Since the STAR mapper assigns reads to a combined reference database comprising both human and viral reference sequences, ends of paired-end reads whose inserts span the breakpoint of a chimeric transcript will be aligned to different reference sequences. These discordant read pairs are reported by STAR during read mapping (see above) and can further be filtered by mismatch score or sequence complexity in order to yield a high-confidence list of chimeric transcripts.

### Generation of homologous regions

**Figure 1:**
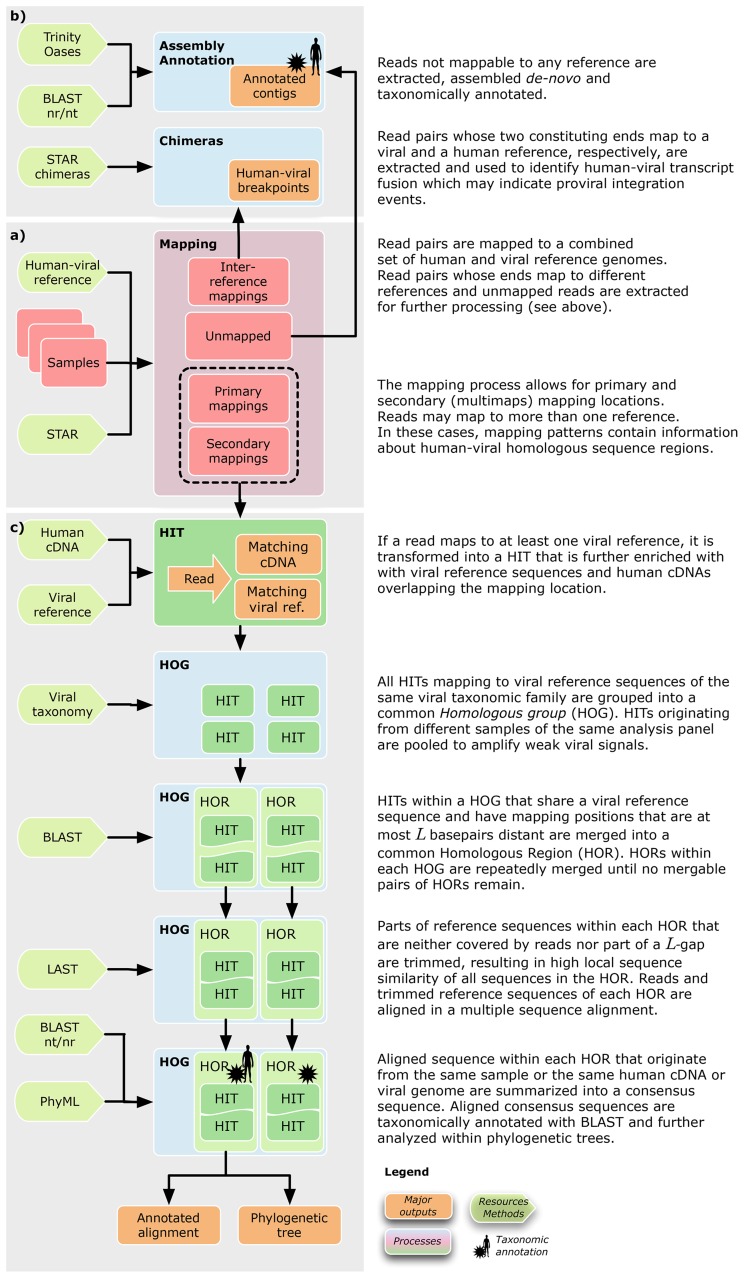
Virana's approach to identifying viral transcripts in human tumors. a) Transcriptome sequence samples are first mapped to a combined set of human and viral reference sequences in a splicing-aware fashion. b) Unmapped or discordantly mapped read pairs are further processed by assembly methods to detect novel viruses or transcript chimeras that may indicate proviral integration events. c) Reads mapping to one or more viral genomes (HITs) are analyzed in an integrated fashion by considering human homologous mapping locations and viral taxonomies. This process results in a number of homologous regions (HOR) for each viral family. HORs are represented as multiple sequence alignments incorporating a wealth of sequence information. Alignments are further enriched by taxonomic annotations and phylogenetic analyses.

A distinguishing feature of Virana is its ability to automatically reconstruct the homologous context of reads that map to both viral and human reference sequences. This homologous context is constructed in four steps:

1. First, reads that map to at least one viral reference are extracted from the mapping together with their primary (highest alignment score) and secondary (up to ten ranks of alignment scores below the highest score) mapping positions (see Figure 1). Since viruses of the same taxonomic family often exhibit significant sequence similarity, reads that map to one family member often also map to related family members as well as to homologous loci in the human reference. Based on these primary and secondary mapping locations, Virana obtains overlapping human reference transcripts, viral genomic references, and viral taxonomic information pertaining to the location. For each sequence read, information obtained in this manner is collected in a data structure denoted as HIT. HITs originating from the same analysis panel are pooled for further analysis.
2. Second, pooled HITs originating from the same analysis panel are assigned to viral taxonomic families based on the viral genomic references they refer to (see Box 1 Algorithm 1). Sets of HITs assigned to the same viral taxonomic family are denoted as the *homologous group* (HOG) of that family. The same HIT may, in principle, be assigned to several HOGs.
3. Third, since reads and references generally share local rather than global sequence similarity, sequences in HOGs cannot conveniently be aligned in a multiple sequence alignment. This circumstance considerably complicates interpretation of homologous relationships between multiple reads and references. Virana therefore applies a three-step greedy clustering approach to split HOGs into manageable and alignable clusters denoted as homologous regions (HORs, see Box 2 Algorithm 2):
  3a The set of all reads within a HOG is re-aligned to the set of all references (human reference transcripts and viral reference genomes) within the HOG using a highly sensitive BLASTN [62] alignment (word size 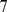). Since all possible mapping locations are required for further processing, BLAST is configured for high permissiveness (E-value 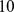).
  3b Each HIT is assigned to a singleton cluster. Clusters containing reads that map to the same reference are merged if their reference mapping locations (as determined by BLASTN) are less or equal than 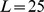 basepairs apart (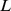-gaps). Optimal values for 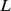 are determined empirically, see Section “Estimation of required sequencing coverage for detection of a homologous region” for a robustness analysis. Merging continues until the number of clusters converges. Subsequently, all clusters with fewer than an empirically chosen cutoff of 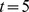 reads are discarded in order to remove spurious hits. After filtering, each remaining cluster represents a candidate HOR. Since cluster membership is defined by reads mapping to common references, each pair of references within the candidate HOR shares one or more regions of high local sequence similarity (e.g., the loci the read mapped to) connected by 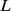-gaps.
  3c For each HOR, parts of reference sequences that are neither covered by a read mapping location nor by an 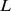-gap between read mapping locations are trimmed.
4. Last, due to the high mutual similarity of sequences within trimmed HORs, sequences within each HOR are now amenable to sequence alignment against the longest reference sequence within that HOR using LASTZ, the successor of BLASTZ [63]. The resulting star-shaped multiple sequence alignment is then used for construction of per-sample (for reads) and per-gene (for human reference transcripts) consensus sequences. Aligned consensus sequences retain information on non-consensus nucleotides due to the usage of IUPAC ambiguous nucleotide codes. Consensus sequences can then be manually inspected in order to determine single nucleotide permutations and indels up to length 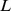 that distinguish sequence reads, viral references, and human reference transcripts.

**Algorithm 1.** Construction of homologous groups.

 
**Data**: Reads

 
**Result**: Homologous groups

 Initialise: homologous_groups;

 
**for** *read in mapping* **do**

  obtain all mapped references of read;

  
**if** *mapped against viral reference* **then**

   Initialize: read_hit;

   add read to read_hit;

   
**for** *viral reference the read maps to* **do**

    add alignment positions of viral reference to     read_hit;

   
**end**

   
**for** *human cDNA reference the read maps to* **do**

    add alignment positions of cDNA to read_hit;

   
**end**

   
**for** *viral reference of a viral taxonomic family thus      added* **do**

    add read_hit to the homologous group for that     family;

   
**end**

  
**end**

 
**end**

**Algorithm 2.** Construction of homologous regions.

**Data**:Homologous groups

**Result**: Homologous regions

Initialise: homologous_regions;

**for** *homologous_group in homologous_groups* **do**

 Initialize: homologous_regions;

 
**for** *read_hit in homologous_group* **do**

  
**for** *homologous_region in homologous_regions* **do**

   
**if** *read_hit and homologous_region share a reference* **then**

    
**if** *alignment positions of shared reference are      within l basepairs* **then**

     merge read_hit into homologous_region;

     extend all reference aligment positions within      homologous_region;

    
**end**

   
**end**

  
**end**

 
**end**

 
**if** *read_hit not merged* **then**

  Initialize: homologous_region;

  merge read_hit into homologous_region;

  add homologous_region to homologous_regions;

 
**end**

**end**

**while** *mergeable_pair_of_homologous_regions exists* **do**

 
**for** *pair_of_homologous_regions in homologous_regions* **do**

  
**if** *pair_of_homologous_regions shares a reference* **then**

   
**if** *alignment positions of shared reference are within l     basepairs* **then**

    merge pair_of_homologous_regions;

    extend all reference aligment positions within     new homologous_region;

   
**end**

  
**end**

 
**end**

**end**

Consensus sequences can be further processed by phylogenetic analyses. For generating phylogenies, Virana employs the software PhyML [64] following the maximum likelihood approach and using default parameters recommended by the HIV sequence database (http://hiv.lanl.gov, GTR model of nucleotide substitution, transition/transversion ratio: 4, gamma shape parameter: 1, number of substation rate categories: 4, approximate Likelihood Ratio Test (aLRT) using SH-like supports where applicable). We note that the topology of the phylogenetic trees constructed in this manner is stable with regard to the model choice; while more complex model parameters may yield better likelihoods in some instances, these differences do not influence interpretation of our results.

### Taxonomic annotation

In this study, we additionally compare consensus sequences of aligned HOGs as well as *de-novo* assembled sequence contigs to nucleotide (NCBI NT) and protein (NCBI NR) reference archives in order to assign transcripts to a taxonomic origin. To this end, we employ several BLAST [58] search strategies (BLASTN, BLASTX, and TBLASTX) with sensitive word sizes (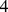, 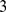, and 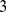, respectively). TBLASTX bypasses synonymous mutations during similarity search and is particularly suited for detecting functionally conserved homologs. This approach is therefore recommended for discovering remote similarities [65] and is widely used in environmental metagenomics [66]. A permissive E-value threshold of 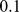 is used for all comparisons in order to reduce the possibility of missing true viral hits. For each query transcript and search strategy, the three highest-scoring reference sequences are extracted from the BLAST results. Subsequently, descriptions, taxonomic information, and available gene annotations for high-scoring reference hits are pooled and query transcripts are assigned a putative viral, human, or ambiguous origin based on the pooled information. In order to limit the search space of the computationally intensive TBLASTX procedure, we constrain the allowed taxonomic origin of reference sequences to only viral (NCBI taxon ID 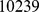) or human (NCBI taxon ID 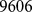) hits while excluding artificial sequences (NCBI taxon ID 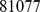) using the NCBI database query “(((txid10239 [ORGN]) OR (txid9606 [ORGN]) OR (human [ORGN])) NOT (txid81077 [ORGN]))”.

### Estimation of read mapping sensitivity

We quantify the ability of our novel method Virana and the related methods RINS [45] and CaPSID [46] at detecting diverged viral transcripts among human sequence data by employing a recently published validation data set [46]. This data set consists of a negative control background set of reads simulated from the human reference genome that is spiked with four sets of 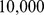 reads simulated from 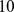 viral reference genomes. Nucleotide positions within reads of each of the four viral spike-in data sets are mutated randomly independently and uniformly with a set-specific probability 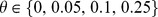 before being merged with the background data set. The set of viral reference sequences represents 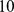 different viral families that infect plants (Cherry green ring mottle virus, Cestrum yellow leaf curling virus, Elm mottle virus, East African cassava mosaic virus), birds (Gallid herpesvirus 1), insects (Cotesia congregata bracovirus), bacteria (Guinea pig Chlamydia phage), amphibians (Frog adenovirus 1), and mammals (Rat coronavirus Parker, Banna virus).

All five data sets (non-spiked human negative control and four human-viral spike-in sets) are analyzed by Virana, RINS, and CaPSID using identical reference sequences as described in Section “Reference genomes”. Sensitivity (fraction of correctly identified viral reads among all viral reads) and specificity (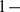 fraction of falsely identified human reads among all human reads) of viral read detection are determined for each method and data set. Analyses are performed with either default parameters (Virana), parameters published in the original validation data set (CaPSID), or settings adapted by us in order to maximize sensitivity (RINS: minimal contig length decreased to 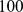, read lengths and insert size distributions according to input data).

Since all methods map to the same complete viral reference set, reads from a particular viral genome of the validation data set may be distributed across several closely related reference genomes, all of which may be considered valid mappings. For this reason, we added post-processing steps to CaPSID and RINS and performed this validation on the level of viral taxonomic families rather than on the level of single viral species. We note, however, that results of all tested methods including Virana retain information on single viral species throughout the analysis. In particular, sensitivity and specificity of the methods change only minimally if data is analyzed on the single species level.

### Analysis of human-viral homologous and chimeric transcripts

Analysis of the human-viral homologous regions and chimeric transcripts based on simulated read data (see Section “Simulated sequencing data”) was conducted by configuring CaPSID, RINS, and Virana analogous to the previous section. For the validation of fusion transcript detection, the number of true positives is set to the number of all reads originating from the human-viral fusion transcript. Since all detection methods in this validation are configured to only report reads mapping to the viral part of the fusion transcript, sensitivity estimates are scaled down equally for all methods in this particular validation. Analysis of discordant read ends in order to detect the origins of chimeric transcripts was performed as described before (see Section “Detection of chimeric transcripts”).

### Estimation of required sequencing depth

Expanding on related work [34], [35], we quantify the theoretical sensitivity of Virana by estimating the number of viral transcripts per cell that are required for achieving a certain minimal sequencing coverage at a probability of at least 95%. Based on human genome annotations obtained from UCSC, we determined an average length of human coding sequences (CDS) of 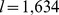 bp. By conservatively assuming that an idealized cell contains 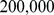 mRNAs [34] of average length 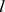 fragmented at 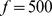 bp as a result of library preparation, an expected number of 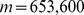 cDNA fragments are generated per cell. For a given viral transcript of length 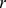 and a viral transcript abundance 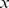 per cell, we expect a number of 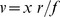 viral transcript fragments. Assuming a theoretical, unbiased sequencing process, the probability of sequencing a viral transcript fragment among the overall 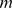 transcript fragments is 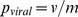. Given a single-end read length of 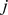, a number 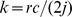 reads are required to achieve a sequence coverage 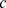 of that viral transcript. The probability 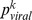 of observing at least 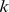 reads during sequencing with a sequencing depth 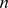 is specified by the cumulative binomial distribution function with parameters 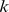, 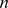 and 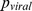. Due to numerical instabilities of computing the cumulative binomial distribution for large values 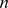, we exploit the Central Limit Theorem and estimate 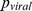 by the Camp-Paulson normal approximation to the binomial distribution. This approach has a negligible approximation error of 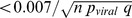, where 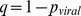 [67]. Our approach further depends on successfully reconstructed homologous regions, each requiring an empirically determined minimum number of 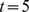 transcripts separated by no more than 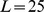 base pairs.

Although the probability 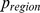 of a homologous region being successfully constructed from viral transcripts at a given sequence coverage can be derived analytically for a special case [68], this solution neither considers edge effects occurring for small transcripts nor takes into account the distribution of insert sizes of paired-end reads. We therefore approach the problem empirically by *in silico* simulation of paired-end reads that are assigned randomly independently and uniformly to transcripts of different lengths and at varying coverages. This simulation process addresses the aforementioned confounding factors by considering transcript boundaries and sampling insert sizes from a normal distribution parametrized according to neuroblastoma sequence data employed in this study (see Section “Library preparation and sequencing”). An mean estimator for 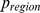 and its standard error 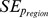 were derived by averaging the success rates of homologous region constructions across 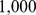 simulations for each transcript length, read length, region linkage, and read coverage.

### Availability

All sequence data generated in this study are publicly available in the European Nucleotide Archive (ENA) at study accession number PRJEB4441. Software implementations of our method and all validation procedures are available at http://mpi-inf.mpg.de/∼sven/virana.

## Results

This study presents a novel approach to detecting viral transcripts in human tumor transcriptomes. In contrast to related approaches such as RINS and CaPSID that rely on subtracting reads homologous to human transcripts from the analysis, our novel method Virana assigns sequence reads to a combined human-viral reference database without discarding homology information (see Figure 1). By employing a particularly fast and sensitive read mapper, Virana gains sensitivity at discovering highly divergent and chimeric viral transcripts. In addition, this configuration allows for exploitation of multimaps (e.g., sequence reads mapping to several reference genomes with varying mismatch rates) to discover the homologous context of sequence reads with regard to viral and human reference sequences. Last, Virana employs chimeric alignments as well as *de-novo* assembly of unmapped sequence reads followed by taxonomic annotation in order to discover proviral integration events and novel viruses, respectively.

### Detection of divergent viruses

**Figure 2:**
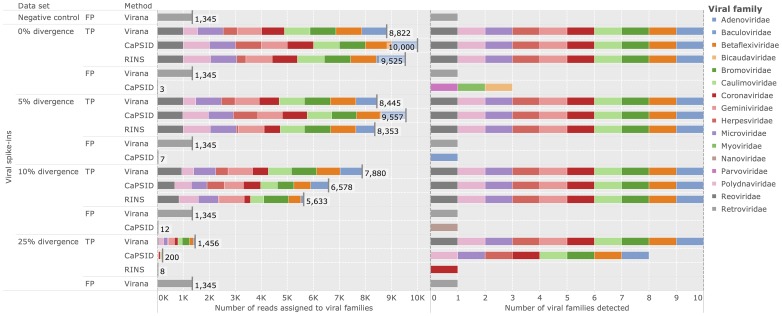
Detection of divergent viruses. Performance comparison of Virana, CaPSID, and RINS at detecting viral reads at different rates of simulated sequence divergence among a background set comprising human genomic reads. The background set without any spike-ins of viral reads serves as negative control. Left panel: stacked bars represent absolute numbers of detected reads grouped by sequence divergence, correctness of classification (TP: true positive, FP: false positive), and detection method. Falsely classified reads not assigned to any of the viral families present in the validation are labeled as false positives (FP). Colored segments indicate to which viral families the reads were assigned. Each condition allowed for the correct detection of up to 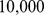 reads. Right panel: color coded markers for each condition and detection method indicating which viral families were identified. A maximum number of 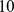 viral families could be correctly identified in each condition.

In order to compare Virana and the two subtractive approaches CaPSID and RINS in a controlled environment we rely on a previously published simulated data set consisting of a negative control data set free of viral reads, here denoted as background set. The background set is used to construct four additional validation data sets spiked with viral reads at increasing rates of sequence divergence (0%, 5%, 10%, 25%, see Materials and Methods). Performance is quantified in terms of sensitivity and specificity (see Materials and Methods). Applying all three viral detection methods on the validation data sets reveals comparatively high rates of correctly detected viral reads for CaPSID and RINS at low sequence divergences between 0% and 5%. Specifically, the two subtractive methods achieve 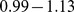 fold higher sensitivities compared to Virana (sensitivities of 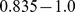 versus 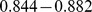 for subtractive approaches and Virana, respectively, see Figure 2). In contrast, Virana substantially surpasses subtractive approaches at higher rates of viral sequence divergence (10–25%), offering comparatively stable sensitivities between 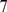-fold and 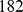-fold higher than Capsid and RINS, respectively (sensitivities of 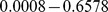 versus 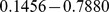 for subtractive approaches and Virana, respectively, see Figure 2, left panel). Notably, while subtractive approaches fail to identify 20–90% of viruses in settings of high sequence divergence, Virana is the only approach able to reliably detect the full set of viruses in all validation scenarios (see Figure 2, right panel). As a result of Virana's ability to detect human-viral transcript homologs, reads originating from several human endogenous retroviruses (HERVs) that are part of the human reference genome but technically also belong to the viral family *Retroviridae* are detected in validation data at all levels of sequence divergence. Since the detected HERV reads originate from the human rather than from the viral part of the validation data, these reads classified as false positive (FP) hits for the purpose of this validation. As a result of this artifact, Virana exhibits a slightly lowered specificity compared to subtractive approaches (0.99985 versus 1.0 for Virana and CaPSID/RINS, respectively). However, we note that HERV reads are correctly classified by Virana during homologous region construction and by optional BLAST-based taxonomic annotation. These reads can therefore be safely and automatically ignored in subsequent analyses if HERV expression is of no interest to the researcher.

**Figure 3:**
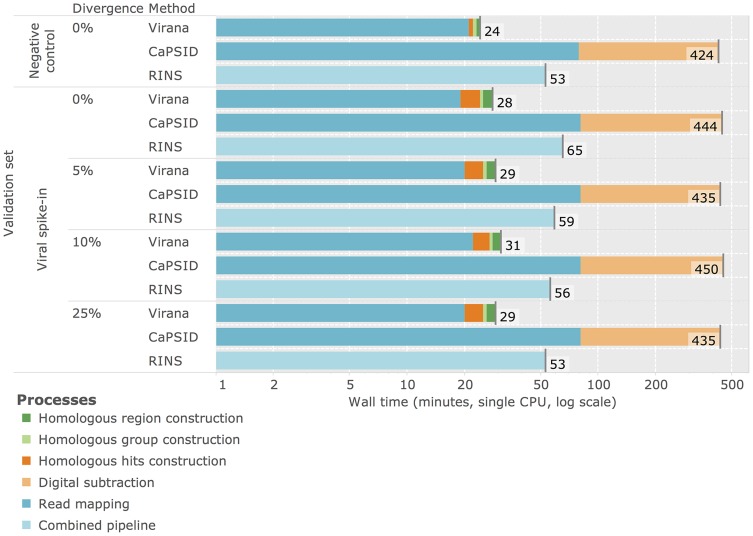
Time required for data analysis. Cumulative time in minutes required for analysis of the divergence validation set. Times are reported for the negative control without viral spike-ins as well as for four mixed data sets consisting of negative control background set with viral spike-ins at different divergence rates. Segments within bar plots represent different analysis processes employed by the three viral detection methods Virana, CaPSID, and RINS. All measurements are based on a single CPU Intel(R) Xeon(R) E5-4640 clocked at 2.40 GHz.

In spite of the involved construction process of homologous regions, Virana is fastest among the three viral detection approaches, requiring only about half an hour per sample analyzed. In contrast, RINS and CaPSID require two to 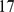 times longer per sample, respectively (see Figure 3). Interestingly, the majority of time spend by CaPSID is lost on subtraction, indicating that this step is a limiting factor of subtractive approaches. We note than reported times are based on analyses using a single compute core. Since all evaluated methods benefit from multithreading, dedicating additional compute cores to the analysis allows for further reduction in processing time.

### Detection of low-coverage, homologous, and chimeric viral transcripts

Having established Virana's ability to detect reads sampled at comparatively high coverage from viral genomes with low or no human-viral sequence similarity, we next test the sensitivity of the viral detection methods in a more challenging scenario involving gene regions of animal viruses that have close human homologs and are sampled at low sequencing coverages. Three such human-viral homologs are used in the analysis: V-ABL of the acutely transforming retrovirus A-MuLV, Bo17 of herpesvirus BoHV-4 (a model virus for oncogenic gammaherpesviruses such as EBV and KSHV and implied in several animal cancers [69]) and *gag* of HERV-K(HML2)22I, a class of human endogenous retroviruses associated with some forms of breast cancer [70]). Validation is based on simulated sequencing data and split into two scenarios (see Materials and Methods for details). Within the first scenario, simulated sequencing reads are sampled directly from human-viral homologs while in the second scenario reads are generated from artificial fusion transcripts that each involve one of the three homologs fused to the human TP53 proto-oncogene. The resulting human-viral fusion transcripts mimic transcriptional signals indicating retroviral integration or homologous recombination of viral DNA next to a human gene which may result in activation of the latter by insertional mutagenesis.

**Figure 4:**
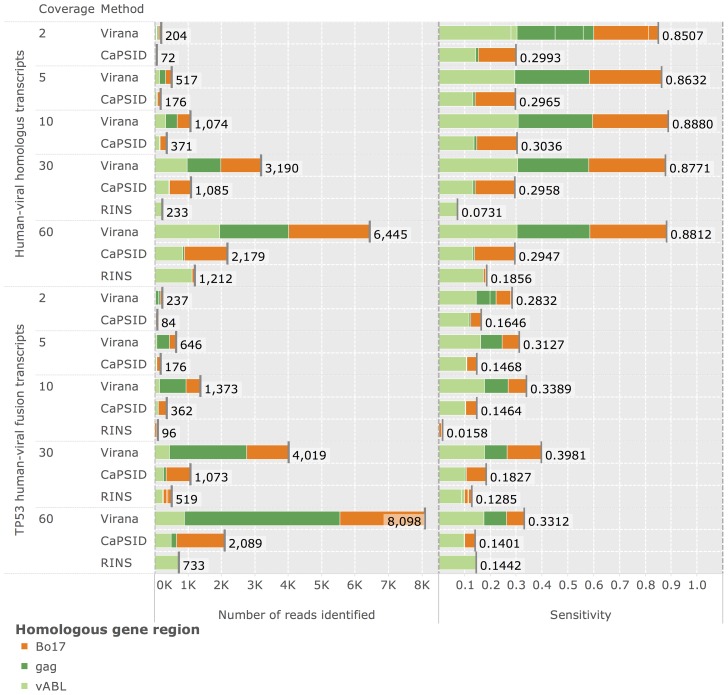
Detection of low-coverage, homologous, and chimeric viral transcripts. Displayed are performances of Virana, CaPSID, and RINS at detecting the three human-viral homologous gene regions Bo17, gag, and vABL. Performance is quantified in terms of sensitivity (right panel) and absolute number of reads correctly identified (left panel) at differing sequencing coverages (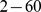 fold). Methods are validated at detecting both isolated gene regions (upper part) as well as at detecting human-viral fusion transcripts involving each of the three gene regions fused to the human TP53 proto-oncogene (lower part). Specificity of detection is 1.0 (100%) for all detection methods (not displayed).

We apply the viral detection methods Virana, CaPSID, and RINS on these two validation data sets in order to evaluate sensitivity at detecting viral genes that are similar to human factors either due to natural sequence homology or due to gene fusions. Performance is quantified by detection sensitivity, specificity, as well as by the absolute number of reads correctly detected. While all methods performed at a perfect specificity of 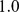, only Virana detects viral transcripts at all coverages and with two to three-fold higher sensitivities compared to competing methods (Figure 4). In particular, sequence reads originating from endogenous retroviruses were almost always subtracted from the analysis by RINS and CaPSID. In addition, RINS seemed to be confounded by low sequencing coverage, a fact most probably resulting from its heavy reliance on *de-novo* transcript assembly. Subsequent analysis of discordantly mapped read pairs by Virana (see Materials and Methods) correctly identified the TP53 gene as fusion partner of both V-ABL and Bo17, indicating that detection of human-viral chimeras is reliable even at low twofold coverage. Due to the repeat nature of the HERV-K sequence in the human genome and the resulting re-occurrence of HERV-K homologs at multiple loci in the human reference it was not possible to unambiguously identify the fusion partner of the HERV-K *gag* gene.

### Estimation of optimal sequencing depth

**Figure 5:**
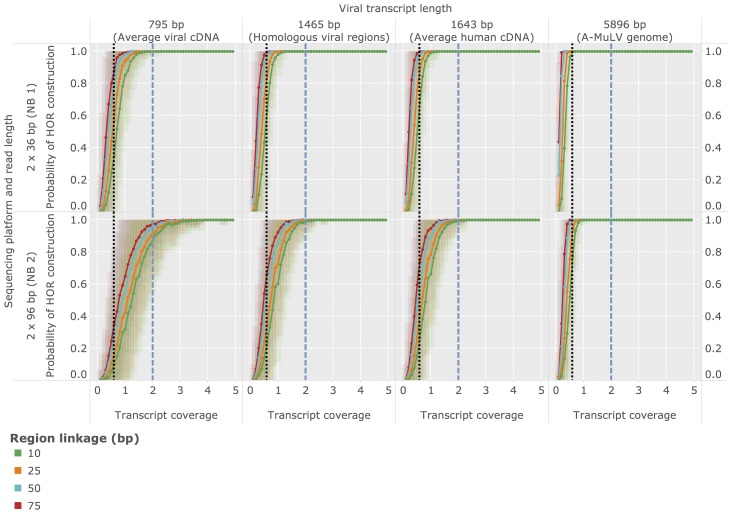
Estimation of required sequencing coverage for detection of a homologous region. Probability of successful region construction by Virana depending on the lengths of the transcripts being sought, the region linkage parameter 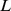, as well as characteristics of the sequencing platform employed. Colored areas represent overlapping standard error bands of the mean, denoting the uncertainties of the estimations. The probability of Virana to detect a homologous region depends on the length of the viral transcript being sought, the linkage parameter 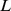 of the homologous region, as well as the transcript coverage and read length of the sequencing platform employed. Given characteristics of the sequencing process applied for NB1 sample panel, an average viral cDNA of length 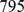 bp requires a minimal transcript coverage of 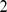 in order to be reliably detected using a linkage parameter of 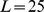 as employed in this study (upper left quadrant, dashed blue vertical line). Technologies affording longer read length as used for the NB2 panel typically also afford higher sequencing depths. However, at a fixed coverage these technologies generate a more highly fragmented region linkage due to a smaller number of longer reads, resulting in lower probability of generating contiguous homologous regions (lower left quadrant). Lower transcript coverage is sufficient for longer transcripts transcribed from a complete A-MuLV genome (upper right panel, dotted black vertical line) or smaller values of the region linkage parameter 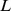.

**Figure 6:**
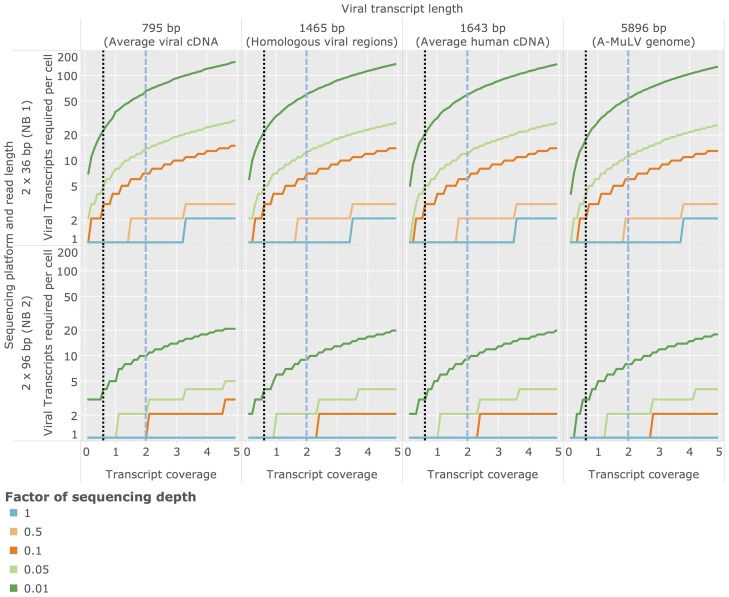
Estimation of required cellular transcript abundances for achieving a given transcript coverage. Sequencing coverage of viral transcripts is depending on the average number of transcript copies per cell in the sequenced sample, on the length of the viral transcript being sought, and on characteristics of the sequencing process. In order to better visualize the optimal sequencing depth required for detection of viral factors, we estimated the required number of transcript copies per cell for different sequencing depths. These sequencing depths are expressed as factors relative to the depths employed for the NB1/NB2 panel generated in this study (which are here reported as a relative sequencing depth of 1).

Due to a variety of factors (see Discussion) human tumor viruses often replicate at very low levels within the infected cell. Determining the required sequencing depth for detecting viral transcripts present at specific cellular abundances is therefore crucial for planning transcriptome experiments designed to identify tumor viruses. Based on statistical arguments and average mRNA sizes (see Materials and Methods), we inferred the minimal abundances of viral transcripts required in an average cell required for detection depending (1) on the length of the transcript being sought and (2) on the sequencing depth employed in the experiment. Here we report results for an average viral cDNA-transcript (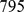 bp), an average viral transcript region analyzed in the validation of human-viral homologs (Bo17 and vABL, 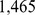 bp, see previous section), an average length human CDS (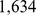 bp), and the genome size of a small tumor virus (A-MuLV, 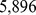 bp). Based on these estimates and given an average sequencing depth as employed in the NB1 analysis panel, Virana requires a minimum twofold sequence coverage of an average viral cDNA transcript in order to detect the transcript within a homologous region with 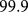
% probability (Figure 5, upper left quadrant, dashed blue vertical line). This sequence coverage is produced with 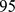
% probability if at least one viral transcript is present per cell, on average (Figure 6, upper left quadrant, dashed blue vertical line). The number of viral transcripts per cell required for detection is inversely related to transcript length and sequencing depth, in principle: at a transcript length corresponding to a small viral genome (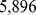 bp) and a per-sample sequencing depth of 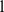
% of the sequencing depth generated in the NB1 panel, a transcript coverage of 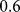 and at least 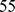 viral transcripts per cell are required for reliable detection (Figure 6, upper right panel, dotted black vertical line).

### Analysis of positive and negative experimental controls

In order to evaluate Virana on experimental data we conducted an analysis of several positive and negative control samples with a cumulative size of 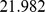 Gbp. The negative control sequencing data originates from a normal brain transcriptome that is suitable as a control for neuroblastoma data. Positive controls span a range of cancer transcriptomes that are associated with several viral cofactors such as a hepatocellular carcinoma (HCC) cell line with proviral integration of Hepatitis B virus, a cervical squamous cell carcinoma (ceSCC) and two HeLa cell line samples with associated human papillomavirus (HPV), and a Ebstein-Barr virus (EBV) positive B-cell lymphoma (BCL).

**Figure 7:**
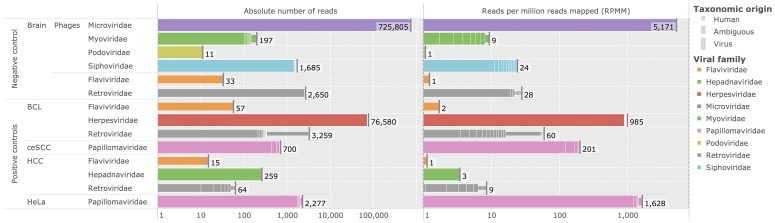
Overview of identified homologous regions in positive and negative experimental controls. Left panel: cumulative numbers of reads assigned to viral taxonomic families (log-scale). Each bar represents a homologous group (HOG) colored according to viral taxonomic family. Bars comprise several segments, each representing a homologous region (HOR). Heights of segments indicate the putative origin of reads assigned to this region (human, viral, or ambiguous). Viral families of bacteriophages are marked accordingly. Right panel: Analogous to left panel, but the lengths of bars represent relative rather than absolute abundances quantified in cumulative reads per million reads mapped (RPMM).

As displayed in Figure 7 (upper part), analysis of the brain negative control sample demonstrates that viral transcription is ubiquitous even in normal (non-cancerous) samples. Specifically, several bacteriophages of the taxonomic families *Microvirodae*, *Myoviridae*, *Podoviridae*, and *Siphovoridae* indicate sample contamination with bacteria as well as technical spike-ins (http://res.illumina.com/documents/products/technotes/technote_phixcontrolv3.pdf). Remarkably, the Coliphage phi-X174 genome of the family *Microvirodae* could be fully assembled by Virana's homologous region construction, yielding a single fragment of 99% sequence identity and 100% coverage compared to the phi-x174 reference genome. In addition, several retroviral and flaviviral hits at low abundances of 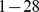 reads per million reads mapped (RPMM) highlight human factors such as HERV-Ks (endogenous retroviruses) as well as human proto-oncogenes SRC/ABL and DNAJC14/RP11 that have close homologs in the viral families *Retroviridae* and *Flaviviridae*, respectively. The taxonomic ambiguity of these regions is automatically identified during Virana's homologous region construction and confirmed by optional BLAST-based annotation compared to NCBI nt and nr databases (as indicated by thinner bars in Figure 7).

Analysis of positive control samples resulted in 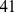 homologous regions (HORs)spanning five viral families (see Figure 7, lower part). Viral cofactors associated with each of the cancer samples are correctly recovered at a high dynamic range of read abundances between 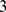 RPMM (HCC with integrated HBV provirus) and 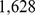 RPMM (HeLa cell line associated with HPV18). In addition, several viral fragments were successfully reconstructed within HORs of the positive control samples, such as a 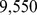 bp long EBV segment containing latency-associated factors EBNA 3b, 3c, and 4a (80% sequence identity with the wild type genome) as well as a 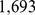 bp long HBV fragment containing the oncogenic HBV-X gene (98% sequence identity compared with Hepatitis B virus isolate HK1476). Similar to results on the negative control brain sample, several HORs with lower abundances assigned to the taxonomic families *Retroviridae* and *Flaviviridae* represent human-viral sequence homologies that are automatically flagged to be of ambiguous taxonomic status by Virana.

Interestingly, the HCC sample was also investigated in recent work focusing on detecting viral integration events [52]. In this recent study, the authors confirmed one integration event by Sanger sequencing while alluding to two additional events still awaiting experimental validation. By analyzing discordantly mapped read ends, Virana could correctly identify all three HBV fusion events involving human genes TRRAP (11 read pairs), ZNF48 (11 read pairs), and PLB1 (6 read pairs) as part of the primary mapping procedure.

### Analysis of neuroblastoma samples

**Figure 8:**
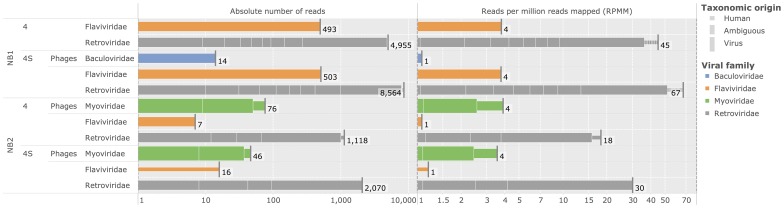
Overview of identified homologous regions in neuroblastoma samples. Left panel: cumulative numbers of reads assigned to viral taxonomic families (log-scale). Each bar represents a homologous group (HOG) colored according to viral taxonomic family. Bars comprise several segments, each representing a homologous region (HOR). Heights of segments indicate the putative origin of reads assigned to this region (human, viral, or ambiguous). Viral families of bacteriophages are marked accordingly. Right panel: Analogous to left panel, but the lengths of bars represent relative rather than absolute abundances quantified in cumulative reads per million mapped (RPMM).

Deep-sequencing of 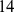 neuroblastoma samples on two sequencing platforms yielded 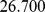 Gbp (NB1) and 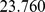 Gbp (NB2) of mapped read pairs (including multimaps), respectively (see Table 2). While samples were sequenced independently and marked with unique identifiers to allow for sample tracking at each step of the analysis, reads from each sample panel and each tumor stage (4 or 4S) were pooled for analysis. Processing the pooled sample panels with Virana resulted in 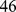 homologous regions representing four viral families (see Figure 8). All HORs were associated with low relative read abundances of 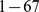 RPMM compared to confirmed viral signatures of experimental positive controls (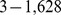 RPMM, see Figure 7). Several homologous regions assigned to bacteriophage viral families *Baculoviridae* and *Myoviridae* are attributable to sample contamination.

**Table 2:** Mapping rates.

Panel	Source	Sample ID	Pairs mapped	Both ends mapped	Uniquely mapped	Depth (Gbp)
POS	HeLa	15	94.900%	94.900%	68.422%	0.127
POS	ceSCC	16	90.803%	90.803%	69.561%	0.264
POS	ceSCC	17	96.629%	96.629%	73.921%	0.075
POS	BCL	18	91.612%	91.612%	63.528%	6.424
POS	HCC	19	94.693%	94.693%	73.500%	14.924
NEG	Brain	20	95.481%	95.481%	72.515%	11.234
NB1	4	1	95.878%	95.878%	69.422%	2.275
NB1	4	2	96.062%	96.062%	74.342%	1.43
NB1	4	3	96.385%	96.385%	75.938%	1.641
NB1	4	4	95.749%	95.749%	71.012%	1.503
NB1	4	5	95.057%	95.057%	69.203%	2.652
NB1	4	6	94.819%	94.819%	69.856%	2.39
NB1	4	7	96.597%	96.597%	72.107%	1.635
NB1	4S	8	95.952%	95.952%	70.681%	2.093
NB1	4S	9	95.242%	95.242%	74.009%	2.223
NB1	4S	10	96.854%	96.854%	74.756%	1.651
NB1	4S	11	96.819%	96.819%	75.256%	1.668
NB1	4S	12	96.710%	96.710%	74.899%	1.539
NB1	4S	13	95.344%	95.344%	72.326%	2.35
NB1	4S	14	97.110%	97.110%	74.829%	1.65
NB2	4	7	86.225%	86.225%	69.552%	12.243
NB2	4S	13	86.280%	86.280%	72.538%	11.517

Mapping ratios and depths of neuroblastoma (NB), positive control (POS), and negative control (NEG) panels. Mapped reads are relative to the number of sequenced read pairs that have passed quality control. Depths include reads with multiple mapping locations (‘multimaps’).

**Figure 9:**
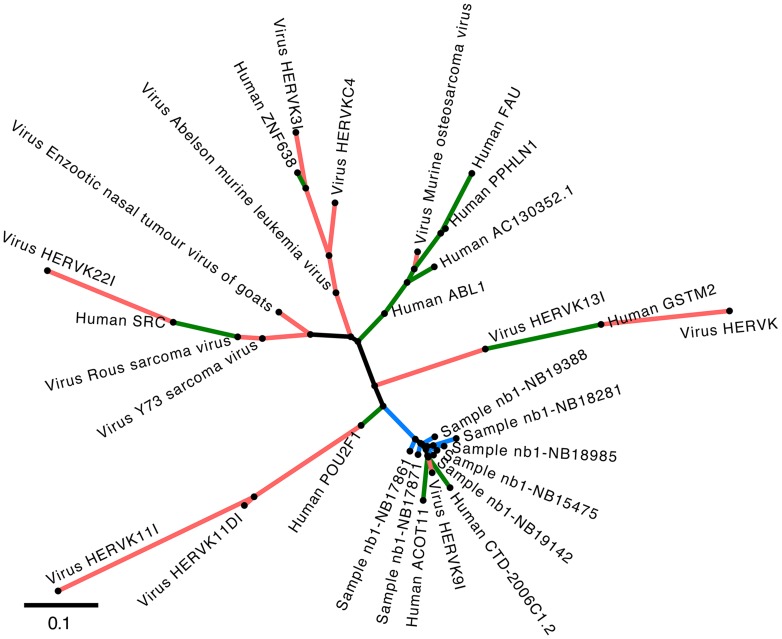
Human-viral phylogeny based on a HOR. Phylogenetic tree of HOR #16 of the NB1 stage 4 panel. Viral reference sequences are indicated with red branches and associated tip labels (‘Virus’) while human factors are labeled with green branches. Blue branches represent consensus sequences of neuroblastoma reads (‘Sample’). The tree was generated by the maximum likelihood approach PhyML using the multiple sequence alignment of the HOR as input (see Materials and Methods). Distances between nodes are quantified as substitutions per site. As can be derived from the tree, neuroblastoma consensus sequences are tightly clustered in close proximity to the endogenous retrovirus HERVK9I and two human factors, thereby unambiguously indicating the human origin of these neuroblastoma reads. Clusters of other sequences represent well known sequence homologies, as for example between human ABL1/SRC genes and acutely transforming retroviruses.

Reads assigned to viral families *Retroviridae* and *Flaviviridae* were determined to originate from either endogenous elements (HERVs) or from human proto-oncogenes that have close homologs in pestiviruses and acutely transforming retroviruses. HORs associated with these viral families were automatically assigned human or ambiguous taxonomic origin by Virana, as indicated by narrower bars in Figure 8. We undertook manual investigation of homologous relationships within each ambiguous HOR by analyzing multiple sequence alignments and phylogenetic trees of the respective regions. These analyses revealed unambiguous clusterings of neuroblastoma sequence reads near human or endogenous factors in all cases (see Figure 9 for an example phylogeny).

No significant differences in viral expression signatures between neuroblastoma 4 and 4S stages could be detected except for HERV-K endogenous retroviruses which display 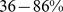 higher abundances in stage 4S (NB1: 56 RPMM, NB2: 28 RPMM) than in stage 4 (NB1: 41 RPMM, NB2: 15 RPMM) neuroblastomas. All reads assigned to homologous regions were further analyzed for evidence of chimeric transcription (see Materials and Methods). While several read pairs with putative chimeric mappings could be identified, all viral chimeric read ends were clustered within low-complexity regions of the viral genomes. Analyses revealed that these putative chimeric mappings represent sequencing errors and low-complexity templates that non-specifically attracted reads of similarly low sequence complexity. No cluster of chimeric reads located at a specifically viral genome location and representing a human-viral breakpoint could be identified.

### Reconstruction of novel transcripts by *de-novo* assembly

**Figure 10:**
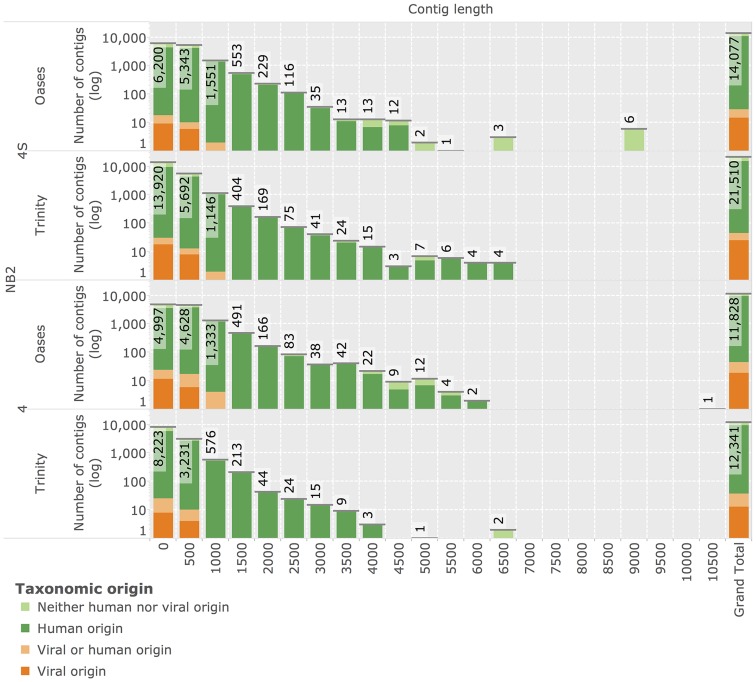
Reconstruction of novel transcripts by *de-novo* assembly. Histograms display lengths of reconstructed sequence contigs assembled from unmapped reads of NB2 stage 4 and stage 4S samples (y-axis in log-space). Two independent assembly methods, Trinity and Oases, were used in the reconstruction. The grand total number of contigs reconstructed within each assembly is displayed in the rightmost column. Reconstructed contigs are annotated with their putative taxonomic origin as inferred by comparison with NCBI nucleotide (nt) and protein (nr) archives using TBLASTX database searches.

In order to identify transcripts of novel viruses that do not map to known references, we generated *de-novo* transcriptome assemblies of all unmapped reads. We applied the two *de Bruijn* graph based assembly methods Oases[61] and Trinity[60] that demonstrated best-in-class performance in recent evaluations [71] on sequencing data of the NB2 panel. This sequencing data is especially amenable to assembly due to its long read length (see Table 1). Assembly resulted in 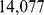 and 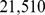 reconstructed neuroblastoma 4S contigs for Oases, and Trinity, respectively (see Figure 10). Assembly of the neuroblastoma 4 sample yielded 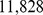 and 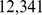 contigs from the same methods. Results of Oases and Trinity assemblies are comparable in terms of contig length. All contigs were subjected to taxonomic annotation using high-sensitivity TBLASTX annotation based on human and viral content of the NCBI nt and nr databases (see Materials and Methods). Overall, 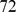 contigs (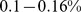 of contigs of any specific assembly) were identified to be of putative viral origin. 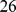 contigs were assigned to bacteriophage references and excluded from further analysis. Based on searches against the full NCBI nr and nt databases followed by manual inspection, all remaining 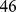 contigs were determined to display higher similarities to bacterial or human sequences than to any viral reference.

## Discussion

Neuroblastoma is a pediatric tumor of the sympathetic nervous system that represents the most common form of cancer in infancy. It is characterized by a striking diversity in biology and clinical behaviour of its subtypes. This heterogeneity as well as supporting epidemiological findings are highly suggestive of infectious cofactors involved in genesis and maintenance of the disease [19], [20]. While several studies utilizing technologies with lower sensitivity compared to our approach have identified human polyomaviruses in neuroblastoma and pediatric embryonal tumors [22]–[24], newer investigations seem to render these associations inconclusive [25]. However, viral commensals of the families *polyomaviridae* and *adenoviridae* are indeed suspected to acquire rare transforming properties as a consequence of viral latency or defective replication [72] and to encode oncogenes [73], [74] whose carcinogenic potential in human is currently investigated [8], [75]. We undertook the first systematic search for known and unknown viruses in transcriptomes of metastatic neuroblastoma by analyzing deep sequencing RNA-Seq data of 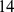 metastatic neuroblastomas from two tumor stages as well as positive and negative experimental controls.

Several high-throughput methods for detecting viral sequence reads among human RNA-Seq data have been developed. Among these methods, PathSeq, CaPSID and RINS are most prominent due to their design as reusable computational pipelines. In this study we selected CaPSID and RINS due to their high performance and public availability and compared their detection performance with that of our novel method Virana. Both CaPSID and RINS follow a subtractive approach, e.g. they separately map input data to viral and human reference sequences and subtract viral read mappings that are similar to the human genome from the analysis. While CaPSID is conceptualised as a generalised framework that supports the subtraction process by means of a database and a web server, RINS features an integrated pipeline that splits input reads into shorter fragments in order to increase mapping sensitivity, followed by transcriptome assembly of putative viral reads into full length transcripts.

Both RNA and DNA viruses may share considerable sequence homology to human factors due to reasons such as lateral gene transfer, oncogene capture, ancestral endogenization, or insertional mutagenesis leading to chimeric transcripts [47]. Such homologous transcripts may display human-viral sequence similarities of 86% (Bovine Herpes virus) and up to 92% (acutely transforming retroviruses). Subtractive approaches silently discard these transcript from the analysis due to their similarity to the human reference genome. In contrast, our novel method Virana follows a radically different approach. Instead of separate mapping to viral and human reference database followed by digital subtraction, Virana undertakes a particularly sensitive read mapping to a combined set of human and viral references. By allowing for multimaps, this mapping strategy facilitates discovery of viral transcripts regardless of their similarity to human factors. Apart from being conceptually simpler by relying on only one mapping step and discarding the subtraction procedure that is both possibly erroneous and computationally costly, this approach empowers the mapper to make informed decisions about relative alignment quality by weighing different human and viral reference positions against each other. As a direct consequence of this increased mapping quality, paired-end reads can be mapped across human and viral references, allowing for detection of human-viral chimeric transcription and proviral integration events.

We quantitatively validated Virana's approach both in settings involving simulated reads as well as in real-world scenarios involving experimental positive and negative controls. In these validations, Virana displays significantly higher detection sensitivities than competing approaches especially at high rates of viral sequence divergence exceeding 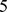
% that are common for tumor viruses [76]–[78]. As a consequence, Virana was the only method able to detect all viral families independent of sequence divergence in the validation data set. In spite of the additional processing undertaken by our method, Virana features between and two and three times faster execution speeds compared to related methods.

Interestingly, viral reads analyzed in the sequence divergence validation originate from a broad array of viral species, only two of which infect mammalian hosts and none of which display significant human-viral sequence homology. As a consequence, this validation favors subtractive approaches by reducing the danger of erroneous subtraction of viral reads that are similar to the human genome. In addition, the sequence divergence validation contained reads sampled at high coverage. However, transcripts of tumor viruses are often expressed at only low cellular abundances and are thus expected to have low sequence coverage. We therefore next validated the ability of viral detection approaches to detect viral transcripts homologous to human factors at varying levels of sequence coverage. Virana, by virtue of not relying on digital subtraction, demonstrated superior sensitivity at this validation both in settings of natural sequence homology as well as in cases of human-viral chimeric transcription. Specifically, Virana was the only method able to detect evidence for all viruses even at low twofold coverages. We observed that both RINS and CaPSID discarded a substantial amount of human-viral homologous transcripts due to their high similarity to the human reference genome, a fact that explains the lower performance of these methods in this validation scenario.

Analysis of positive and negative experimental controls further reveals that Virana is able to detect viral transcripts associated with four types of cancer at a high dynamic range of relative abundances. While Virana displays a slightly reduced specificity in simulated and experimental evaluations, these false positive hits are limited to only two viral families (*Flaviviridae* and *Retroviridae*) that display high sequence similarity to human factors. These hits are additionally annotated with an ambiguous taxonomic origin by Virana. In addition, Virana provides extensive support for investigating such ambiguous viral hits by analyzing the homologous context of putative viral reads in a context of multiple sequence alignments and phylogenies.

In principle, several biological confounding factors may hinder detection of viral transcripts by any sequence-based method. Low concentration and extratumoral location of viral producer cells [8] or selection of growth-autonomous cells in progressed tumors [79] can significantly dilute the number of viral transcripts in a sample. Additionally, known tumor viruses such as high-risk HPV strains, EBV, and MCPyV selectively transcribe their genome during viral latency (HPV: E6/7 [80], [81], EBV: EBNA1/2 [82]–[84], MCPyV: large T antigen [31], [85]), thus generating only low abundances of tens (MCPyV [31]) to hundreds (KSHV [86], EBV [87]) of transcripts per cell. Last, transcription of human oncogenic factors modulated by viral [88] or endogenous [89], [90] retroviral promoters as well as ‘hit-and-run’ mechanisms of viral oncogenesis that imply loss of viral material [91], [92] may predispose cells to transformation without requiring maintenance of viral transcripts.

Our approach aims to counteract these confounding factors by two strategies: first by sequencing neuroblastoma transcriptomes at comparatively high depth in order to detect rare transcripts and second by using several biological replicates at different tumor stages, thus reducing the probability of total loss of viral material from all analyzed samples. Based on statistical estimations concerning Virana's homologous region construction process and the sequencing depth of our experimental data, we can conclude that our approach requires minimal abundances of only two average-length viral transcripts per cell even under adverse conditions such as high viral divergence or extensive human-viral sequence homology. While representing a theoretical sensitivity that may be altered by sequencing biases [93], these copy numbers compare very favorably with related estimates reporting minimal abundances of one to several complete viral genomes per cell [27], .

After applying Virana to several positive control panels of human cancers with known viral cofactors and accurately reconstructing large fragments of viruses that are causally related to the respective tumors, we analyzed neuroblastoma transcriptomes at high sequencing depth and using two different sequencing platforms. Analyses of neuroblastoma transcriptomes resulted in the detection of putative viral transcripts with high local sequence similarity to several viral families. However, automatic taxonomic annotation as well as detailed manual inspection of homologous regions pertaining to these families revealed the human or bacteriophage origin of all transcripts. While we could find differences in the abundance of HERV-K transcripts between neuroblastoma stages 4 and 4S, the causative role of HERV transcription with regard to oncogenesis is currently unclear [94] and, as to our knowledge, only tentative associations with specific cancers have been made as to date [70]. Apart from these tentative differences in HERV-K abundances, no quantitative difference between neuroblastoma stages 4 and 4S could be identified with regard to viral transcription.

In conclusion, our observations provide negative evidence regarding the contested question of putative viral cofactors of metastatic neuroblastoma by suggesting that viruses are unlikely to be frequent cofactors in the maintenance of metastatic neuroblastoma.

## References

[1] Moore PS, Chang Y (2010) Why do viruses cause cancer? Highlights of the first century of human tumour virology. Nat Rev Cancer 10: 878–889. 21102637 10.1038/nrc2961 PMC3718018

[2] Parkin DM (2006) The global health burden of infection-associated cancers in the year 2002. Int J Cancer 118: 3030–3044. 16404738 10.1002/ijc.21731

[3] Sarid R, Gao SJ (2011) Viruses and human cancer: from detection to causality. Cancer Lett 305: 218–227. 20971551 10.1016/j.canlet.2010.09.011 PMC3037427

[4] Schiller JT, Lowy DR (2010) Vaccines to prevent infections by oncoviruses. Annu Rev Microbiol 64: 23–41. 20420520 10.1146/annurev.micro.112408.134019 PMC6264788

[5] zur Hausen H (2006) Infections Causing Human Cancer. Weinheim: Wiley-VCH.

[6] zur Hausen H (2012) Red meat consumption and cancer: reasons to suspect involvement of bovine infectious factors in colorectal cancer. Int J Cancer 130: 2475–2483. 22212999 10.1002/ijc.27413

[7] International Cancer Genome Consortium (2010) Hudson TJ, Anderson W, Artez A, Barker AD, et al (2010) International network of cancer genome projects. Nature 464: 993–998. 20393554 10.1038/nature08987 PMC2902243

[8] zur Hausen H (2009) The search for infectious causes of human cancers: where and why. Virology 392: 1–10. 19720205 10.1016/j.virol.2009.06.001

[9] Javier RT, Butel JS (2008) The History of Tumor Virology. Cancer Research 68: 7693–7706. 18829521 10.1158/0008-5472.CAN-08-3301 PMC3501656

[10] Brodeur GM (2003) Neuroblastoma: biological insights into a clinical enigma. Nat Rev Cancer 3: 203–216. 12612655 10.1038/nrc1014

[11] Maris JM, Hogarty MD, Bagatell R, Cohn SL (2007) Neuroblastoma. The Lancet 369: 2106–2120. 10.1016/S0140-6736(07)60983-0 17586306

[12] Janoueix-Lerosey I, Schleiermacher G, Delattre O (2010) Molecular pathogenesis of peripheral neuroblastic tumors. Oncogene 29: 1566–1579. 20101209 10.1038/onc.2009.518

[13] Kaatsch P (2010) Epidemiology of childhood cancer. Cancer Treat Rev 36: 277–285. 20231056 10.1016/j.ctrv.2010.02.003

[14] D'Angio GJ, Evans AE, Koop CE (1971) Special pattern of widespread neuroblastoma with a favourable prognosis. Lancet 1: 1046–1049. 4102970 10.1016/s0140-6736(71)91606-0

[15] Shuangshoti S, Shuangshoti S, Nuchprayoon I, Kanjanapongkul S, Marrano P, et al (2012) Natural course of low risk neuroblastoma. Pediatr Blood Cancer 58: 690–694. 21922650 10.1002/pbc.23325

[16] Janoueix-Lerosey I, Schleiermacher G, Michels E, Mosseri V, Ribeiro A, et al (2009) Overall genomic pattern is a predictor of outcome in neuroblastoma. J Clin Oncol 27: 1026–1033. 19171713 10.1200/JCO.2008.16.0630

[17] Fischer M, Oberthuer A, Brors B, Kahlert Y, Skowron M, et al (2006) Differential expression of neuronal genes defines subtypes of disseminated neuroblastoma with favorable and unfavorable outcome. Clin Cancer Res 12: 5118–5128. 16951229 10.1158/1078-0432.CCR-06-0985

[18] Roman E, Simpson J, Ansell P, Kinsey S, Mitchell CD, et al (2007) Childhood acute lymphoblastic leukemia and infections in the first year of life: a report from the United Kingdom Childhood Cancer Study. Am J Epidemiol 165: 496–504. 17182983 10.1093/aje/kwk039

[19] Menegaux F, Olshan AF, Neglia JP, Pollock BH, Bondy ML (2004) Day care, childhood infections, and risk of neuroblastoma. Am J Epidemiol 159: 843–851. 15105177 10.1093/aje/kwh111 PMC2080646

[20] Heck JE, Ritz B, Hung RJ, Hashibe M, Boffetta P (2009) The epidemiology of neuroblastoma: a review. Paediatr Perinat Epidemiol 23: 125–143. 19159399 10.1111/j.1365-3016.2008.00983.x

[21] zur Hausen H (2009) Childhood leukemias and other hematopoietic malignancies: interdependence between an infectious event and chromosomal modifications. Int J Cancer 125: 1764–1770. 19330827 10.1002/ijc.24365

[22] Jørgensen GE, Johnsen JI, Ponthan F, Kogner P, Flaegstad T, et al (2000) Human polyomavirus BK (BKV) and neuroblastoma: mechanisms of oncogenic action and possible strategy for novel treatment. Med Pediatr Oncol 35: 593–596. 11107125 10.1002/1096-911x(20001201)35:6<593::aid-mpo22>3.0.co;2-i

[23] Krynska B, Del Valle L, Croul S, Gordon J, Katsetos CD, et al (1999) Detection of human neurotropic JC virus DNA sequence and expression of the viral oncogenic protein in pediatric medulloblastomas. Proc Natl Acad Sci USA 96: 11519–11524. 10500209 10.1073/pnas.96.20.11519 PMC18066

[24] Flaegstad T, Andresen PA, Johnsen JI, Asomani SK, Jørgensen GE, et al (1999) A possible contributory role of BK virus infection in neuroblastoma development. Cancer Research 59: 1160–1163. 10070978

[25] Stolt A, Kjellin M, Sasnauskas K, Luostarinen T, Koskela P, et al (2005) Maternal human polyomavirus infection and risk of neuroblastoma in the child. Int J Cancer 113: 393–396. 15455352 10.1002/ijc.20573

[26] Rohwer F, Edwards R (2002) The Phage Proteomic Tree: a genome-based taxonomy for phage. J Bacteriol 184: 4529–4535. 12142423 10.1128/JB.184.16.4529-4535.2002 PMC135240

[27] Bexfield N, Kellam P (2011) Metagenomics and the molecular identification of novel viruses. Vet J 190: 191–198. 21111643 10.1016/j.tvjl.2010.10.014 PMC7110547

[28] Chang Y, Cesarman E, Pessin MS, Lee F, Culpepper J, et al (1994) Identification of herpesvirus-like DNA sequences in AIDS-associated Kaposi's sarcoma. Science 266: 1865–1869. 7997879 10.1126/science.7997879

[29] Feldhahn M, Menzel M, Weide B, Bauer P, Meckbach D, et al (2011) No evidence of viral genomes in whole-transcriptome sequencing of three melanoma metastases. Exp Dermatol 20: 766–768. 21672032 10.1111/j.1600-0625.2011.01312.x

[30] Arron ST, Ruby JG, Dybbro E, Ganem D, DeRisi JL (2011) Transcriptome sequencing demonstrates that human papillomavirus is not active in cutaneous squamous cell carcinoma. J Invest Dermatol 131: 1745–1753. 21490616 10.1038/jid.2011.91 PMC3136639

[31] Feng H, Shuda M, Chang Y, Moore PS (2008) Clonal integration of a polyomavirus in human Merkel cell carcinoma. Science 319: 1096–1100. 18202256 10.1126/science.1152586 PMC2740911

[32] Lipkin WI (2010) Microbe hunting. Microbiol Mol Biol Rev 74: 363–377. 20805403 10.1128/MMBR.00007-10 PMC2937520

[33] Duncan CG, Leary RJ, Lin JCH, Cummins J, Di C, et al (2009) Identification of microbial DNA in human cancer. BMC medical genomics 2: 22. 19426505 10.1186/1755-8794-2-22 PMC2685141

[34] Feng H, Taylor JL, Benos PV, Newton R, Waddell K, et al (2007) Human transcriptome subtraction by using short sequence tags to search for tumor viruses in conjunctival carcinoma. J Virol 81: 11332–11340. 17686852 10.1128/JVI.00875-07 PMC2045575

[35] Moore RA, Warren RL, Freeman JD, Gustavsen JA, Chénard C, et al (2011) The sensitivity of massively parallel sequencing for detecting candidate infectious agents associated with human tissue. PLoS ONE 6: e19838. 21603639 10.1371/journal.pone.0019838 PMC3094400

[36] Xu Y, Stange-Thomann N, Weber G, Bo R, Dodge S, et al (2003) Pathogen discovery from human tissue by sequence-based computational subtraction. Genomics 81: 329–335. 12659816 10.1016/s0888-7543(02)00043-5

[37] Weber G, Shendure J, Tanenbaum DM, Church GM, Meyerson M (2002) Identification of foreign gene sequences by transcript filtering against the human genome. Nat Genet 30: 141–142. 11788827 10.1038/ng818

[38] Isakov O, Modai S, Shomron N (2011) Pathogen detection using short-RNA deep sequencing subtraction and assembly. Bioinformatics 27: 2027–2030. 21666269 10.1093/bioinformatics/btr349 PMC3137223

[39] Patowary A, Chauhan RK, Singh M, Kv S, Periwal V, et al (2012) De novo identification of viral pathogens from cell culture hologenomes. BMC Res Notes 5: 11. 22226071 10.1186/1756-0500-5-11 PMC3284880

[40] Ma M, Huang Y, Gong Z, Zhuang L, Li C, et al (2011) Discovery of DNA Viruses in Wild-Caught Mosquitoes Using Small RNA High throughput Sequencing. PLoS ONE 6: e24758. 21949749 10.1371/journal.pone.0024758 PMC3176773

[41] Kreuze JF, Perez A, Untiveros M, Quispe D, Fuentes S, et al (2009) Complete viral genome sequence and discovery of novel viruses by deep sequencing of small RNAs: a generic method for diagnosis, discovery and sequencing of viruses. Virology 388: 1–7. 19394993 10.1016/j.virol.2009.03.024

[42] Palacios G, Druce J, Du L, Tran T, Birch C, et al (2008) A new arenavirus in a cluster of fatal transplant-associated diseases. N Engl J Med 358: 991–998. 18256387 10.1056/NEJMoa073785

[43] Wu Q, Luo Y, Lu R, Lau N, Lai EC, et al (2010) Virus discovery by deep sequencing and assembly of virus-derived small silencing RNAs. Proc Natl Acad Sci USA 107: 1606–1611. 20080648 10.1073/pnas.0911353107 PMC2824396

[44] Kostic AD, Ojesina AI, Pedamallu CS, Jung J, Verhaak RGW, et al (2011) PathSeq: software to identify or discover microbes by deep sequencing of human tissue. Nat Biotechnol 29: 393–396. 21552235 10.1038/nbt.1868 PMC3523678

[45] Bhaduri A, Qu K, Lee CS, Ungewickell A, Khavari PA (2012) Rapid identification of non-human sequences in high-throughput sequencing datasets. Bioinformatics 28: 1174–1175. 22377895 10.1093/bioinformatics/bts100 PMC3324519

[46] Borozan I, Wilson S, Blanchette P, Laamme P, Watt SN, et al (2012) CaPSID: A bioinformatics platform for computational pathogen sequence identification in human genomes and transcriptomes. BMC Bioinformatics 13: 206. 22901030 10.1186/1471-2105-13-206 PMC3464663

[47] Butel JS (2000) Viral carcinogenesis: revelation of molecular mechanisms and etiology of human disease. Carcinogenesis 21: 405–426. 10688861 10.1093/carcin/21.3.405

[48] Duffy S, Shackelton LA, Holmes EC (2008) Rates of evolutionary change in viruses: patterns and determinants. Nat Rev Genet 9: 267–276. 18319742 10.1038/nrg2323

[49] Firth C, Kitchen A, Shapiro B, Suchard MA, Holmes EC, et al (2010) Using time-structured data to estimate evolutionary rates of double-stranded DNA viruses. Mol Biol Evol 27: 2038–2051. 20363828 10.1093/molbev/msq088 PMC3107591

[50] Spitz R, Hero B, Ernestus K, Berthold F (2003) FISH analyses for alterations in chromosomes 1, 2, 3, and 11 define high-risk groups in neuroblastoma. Med Pediatr Oncol 41: 30–35. 12764740 10.1002/mpo.10313

[51] Aschoff M, Hotz-Wagenblatt A, Glatting KH, Fischer M, Eils R, et al (2013) SplicingCompass: differential splicing detection using RNA-Seq data. Bioinformatics 29: 1141–1148. 23449093 10.1093/bioinformatics/btt101

[52] Li JW, Wan R, Yu CS, Co NN, Wong N, et al (2013) ViralFusionSeq: accurately discover viral integration events and reconstruct fusion transcripts at single-base resolution. Bioinformatics 29: 649–651. 23314323 10.1093/bioinformatics/btt011 PMC3582262

[53] Jurka J, Kapitonov VV, Pavlicek A, Klonowski P, Kohany O, et al (2005) Repbase Update, a database of eukaryotic repetitive elements. Cytogenet Genome Res 110: 462–467. 16093699 10.1159/000084979

[54] Li H, Handsaker B, Wysoker A, Fennell T, Ruan J, et al (2009) The Sequence Alignment/Map format and SAMtools. Bioinformatics 25: 2078–2079. 19505943 10.1093/bioinformatics/btp352 PMC2723002

[55] Bruno AE, Miecznikowski JC, Qin M, Wang J, Liu S (2013) FUSIM: a software tool for simulating fusion transcripts. BMC Bioinformatics 14: 13. 23323884 10.1186/1471-2105-14-13 PMC3637076

[56] Pruitt KD, Tatusova T, Klimke W, Maglott DR (2009) NCBI Reference Sequences: current status, policy and new initiatives. Nucleic Acids Res 37: D32–6. 18927115 10.1093/nar/gkn721 PMC2686572

[57] Flicek P, Amode MR, Barrell D, Beal K, Brent S, et al (2012) Ensembl 2012. Nucleic Acids Res 40: D84–90. 22086963 10.1093/nar/gkr991 PMC3245178

[58] Maglott D, Ostell J, Pruitt KD, Tatusova T (2011) Entrez Gene: gene-centered information at NCBI. Nucleic Acids Res 39: D52–7. 21115458 10.1093/nar/gkq1237 PMC3013746

[59] Dobin A, Davis CA, Schlesinger F, Drenkow J, Zaleski C, et al (2013) STAR: ultrafast universal RNA-seq aligner. Bioinformatics 29: 15–21. 23104886 10.1093/bioinformatics/bts635 PMC3530905

[60] Grabherr MG, Haas BJ, Yassour M, Levin JZ, Thompson DA, et al (2011) Fulllength transcriptome assembly from RNA-Seq data without a reference genome. Nat Biotechnol 29: 644–652. 21572440 10.1038/nbt.1883 PMC3571712

[61] Schulz MH, Zerbino DR, Vingron M, Birney E (2012) Oases: robust de novo RNA-seq assembly across the dynamic range of expression levels. Bioinformatics 28: 1086–1092. 22368243 10.1093/bioinformatics/bts094 PMC3324515

[62] Altschul SF, Gish W, Miller W, Myers EW, Lipman DJ (1990) Basic local alignment search tool. J Mol Biol 215: 403–410. 2231712 10.1016/S0022-2836(05)80360-2

[63] Schwartz S, Kent WJ, Smit A, Zhang Z, Baertsch R, et al (2003) Human-mouse alignments with BLASTZ. Genome Res 13: 103–107. 12529312 10.1101/gr.809403 PMC430961

[64] Guindon S, Dufayard JF, Lefort V, Anisimova M, Hordijk W, et al (2010) New algorithms and methods to estimate maximum-likelihood phylogenies: assessing the performance of PhyML 3.0. Syst Biol 59: 307–321. 20525638 10.1093/sysbio/syq010

[65] Kunin V, Copeland A, Lapidus A, Mavromatis K, Hugenholtz P (2008) A bioinformatician's guide to metagenomics. Microbiol Mol Biol Rev 72: 557–578. 19052320 10.1128/MMBR.00009-08 PMC2593568

[66] Mokili JL, Rohwer F, Dutilh BE (2012) Metagenomics and future perspectives in virus discovery. Current Opinion in Virology 2: 63–77. 22440968 10.1016/j.coviro.2011.12.004 PMC7102772

[67] Lesch SM, Jeske DR (2009) Some Suggestions for Teaching About Normal Approximations to Poisson and Binomial Distribution Functions. The American Statistician 63: 274–277.

[68] Breitbart M, Salamon P, Andresen B, Mahaffy JM, Segall AM, et al (2002) Genomic analysis of uncultured marine viral communities. Proc Natl Acad Sci USA 99: 14250–14255. 12384570 10.1073/pnas.202488399 PMC137870

[69] Thiry E, Bublot M, Dubuisson J, Van Bressem MF, Lequarre AS, et al (1992) Molecular biology of bovine herpesvirus type 4. Vet Microbiol 33: 79–92. 1336253 10.1016/0378-1135(92)90037-t

[70] Wang-Johanning F, Frost AR, Jian B, Epp L, Lu DW, et al (2003) Quantitation of HERV-K env gene expression and splicing in human breast cancer. Oncogene 22: 1528–1535. 12629516 10.1038/sj.onc.1206241

[71] Zhao QY, Wang Y, Kong YM, Luo D, Li X, et al (2011) Optimizing de novo transcriptome assembly from short-read RNA-Seq data: a comparative study. BMC Bioinformatics 12 Suppl 14: S2. 10.1186/1471-2105-12-S14-S2 PMC3287467 22373417

[72] zur Hausen H (2001) Oncogenic DNA viruses. Oncogene 20: 7820–7823. 11753664 10.1038/sj.onc.1204958

[73] Berk AJ (2005) Recent lessons in gene expression, cell cycle control, and cell biology from adenovirus. Oncogene 24: 7673–7685. 16299528 10.1038/sj.onc.1209040

[74] Eash S, Manley K, Gasparovic M, Querbes W, Atwood WJ (2006) The human polyomaviruses. Cell Mol Life Sci 63: 865–876. 16501889 10.1007/s00018-005-5454-z PMC11136111

[75] Elgui de Oliveira D (2007) DNA viruses in human cancer: an integrated overview on fundamental mechanisms of viral carcinogenesis. Cancer Lett 247: 182–196. 16814460 10.1016/j.canlet.2006.05.010

[76] de Villiers EM, Fauquet C, Broker TR, Bernard HU, zur Hausen H (2004) Classification of papillomaviruses. Virology 324: 17–27. 15183049 10.1016/j.virol.2004.03.033

[77] Simmonds P, Bukh J, Combet C, Deleage G, Enomoto N, et al (2005) Consensus proposals for a unified system of nomenclature of hepatitis C virus genotypes. Hepatology 42: 962–973. 16149085 10.1002/hep.20819

[78] Karlin S, Blaisdell BE, Schachtel GA (1990) Contrasts in codon usage of latent versus productive genes of Epstein-Barr virus: data and hypotheses. J Virol 64: 4264–4273. 2166815 10.1128/jvi.64.9.4264-4273.1990 PMC247892

[79] Voisset C, Weiss RA, Griffiths DJ (2008) Human RNA “rumor” viruses: the search for novel human retroviruses in chronic disease. Microbiol Mol Biol Rev 72: 157–196. 18322038 10.1128/MMBR.00033-07 PMC2268285

[80] Dyson N, M HP, Münger K, Harlow E (1989) The human papilloma virus-16 E7 oncoprotein is able to bind to the retinoblastoma gene product. Science 243: 934–937. 2537532 10.1126/science.2537532

[81] Scheffner M, Werness BA, Huibregtse JM, Levine AJ, M HP (1990) The E6 oncoprotein encoded by human papillomavirus types 16 and 18 promotes the degradation of p53. Cell 63: 1129–1136. 2175676 10.1016/0092-8674(90)90409-8

[82] Young LS, Rickinson AB (2004) Epstein-Barr virus: 40 years on. Nat Rev Cancer 4: 757–768. 15510157 10.1038/nrc1452

[83] Kelly GL, Long HM, Stylianou J, Thomas WA, Leese A, et al (2009) An Epstein-Barr virus anti-apoptotic protein constitutively expressed in transformed cells and implicated in burkitt lymphomagenesis: the Wp/BHRF1 link. PLoS Pathog 5: e1000341. 19283066 10.1371/journal.ppat.1000341 PMC2652661

[84] Klein E, Kis LL, Klein G (2007) Epstein-Barr virus infection in humans: from harmless to life endangering virus-lymphocyte interactions. Oncogene 26: 1297–1305. 17322915 10.1038/sj.onc.1210240

[85] Houben R, Shuda M, Weinkam R, Schrama D, Feng H, et al (2010) Merkel cell polyomavirus-infected Merkel cell carcinoma cells require expression of viral T antigens. J Virol 84: 7064–7072. 20444890 10.1128/JVI.02400-09 PMC2898224

[86] Cornelissen M, van der Kuyl AC, van den Burg R, Zorgdrager F, van Noesel CJM, et al (2003) Gene expression profile of AIDS-related Kaposi's sarcoma. BMC Cancer 3: 7. 12697073 10.1186/1471-2407-3-7 PMC155676

[87] Metzenberg S (1990) Levels of Epstein-Barr virus DNA in lymphoblastoid cell lines are correlated with frequencies of spontaneous lytic growth but not with levels of expression of EBNA-1, EBNA-2, or latent membrane protein. J Virol 64: 437–444. 2152830 10.1128/jvi.64.1.437-444.1990 PMC249123

[88] Coffin JM, Hughes SH, Varmus HE, Rosenberg N, Jolicoeur P (1997) Retroviral Pathogenesis. Retroviruses. Cold Spring Harbor (NY): Cold Spring Harbor Laboratory Press. 21433341

[89] Ono M, Yasunaga T, Miyata T, Ushikubo H (1986) Nucleotide sequence of human endogenous retrovirus genome related to the mouse mammary tumor virus genome. J Virol 60: 589–598. 3021993 10.1128/jvi.60.2.589-598.1986 PMC288930

[90] Tomlins SA, Laxman B, Dhanasekaran SM, Helgeson BE, Cao X, et al (2007) Distinct classes of chromosomal rearrangements create oncogenic ETS gene fusions in prostate cancer. Nature 448: 595–599. 17671502 10.1038/nature06024

[91] Si H, Robertson ES (2006) Kaposi's sarcoma-associated herpesvirus-encoded latencyassociated nuclear antigen induces chromosomal instability through inhibition of p53 function. J Virol 80: 697–709. 16378973 10.1128/JVI.80.2.697-709.2006 PMC1346846

[92] McLaughlin-Drubin ME, Munger K (2008) Viruses associated with human cancer. Biochim Biophys Acta 1782: 127–150. 18201576 10.1016/j.bbadis.2007.12.005 PMC2267909

[93] Fang Z, Cui X (2011) Design and validation issues in RNA-seq experiments. Brief Bioinform 12: 280–287. 21498551 10.1093/bib/bbr004

[94] Bannert N, Kurth R (2004) Retroelements and the human genome: new perspectives on an old relation. Proc Natl Acad Sci USA 101 Suppl 2: 14572–14579. 15310846 10.1073/pnas.0404838101 PMC521986

